# Conspiracies in Academia? Stand Up Against Defamations of Open Access Journals!

**DOI:** 10.3389/ijph.2025.1608614

**Published:** 2025-05-13

**Authors:** Nino Künzli, Christopher Woodrow, Luca Crivelli, Kasia Czabanowska, Olaf von dem Knesebeck, Andrea Madarasova Geckova, Sarah Mantwill, Sunghea Park, Milo Puhan, Ana Isabel Ribeiro, L. Suzanne Suggs

**Affiliations:** ^1^ Swiss Tropical and Public Health Institute (SwissTPH), Allschwil, Switzerland; ^2^ Swiss School of Public Health (SSPH+), Zürich, Switzerland; ^3^ Department of Business Economics, Health and Social Care, University of Applied Sciences and Arts of Southern Switzerland (SUPSI), Lugano, Switzerland; ^4^ Department of International Health, Care and Public Health Research Institute, Maastricht University, Maastricht, Netherlands; ^5^ Institute of Medical Sociology, University of Hamburg, Hamburg, Germany; ^6^ Department of Health Psychology and Research Methodology, Pavol Jozef Šafárik University in Košice, Košice, Slovakia; ^7^ Faculty of Health Sciences and Medicine, University of Lucerne, Lucerne, Switzerland; ^8^ Epidemiology, Biostatistics and Prevention Institute (EBPI), University Zürich, Zürich, Switzerland; ^9^ Epidemiology Research Unit, Institute of Public Health, University of Porto, Porto, Portugal; ^10^ Institute of Communication and Public Policy, Università della Svizzera Italiana (USI), Lugano, Switzerland

**Keywords:** publishing, open access, public health, ranking systems, journal, bibliometry, ethics, fraud

The Open Access publishing model (OA) makes research findings freely and publicly accessible to everybody, including all taxpayers who contribute to publicly funded research. It fundamentally contrasts with the traditional subscription-based model, where science is locked behind paywalls. In the OA model, related costs are instead covered by Article Processing Charges (APC) paid by the authors (Gold OA); in relatively rare cases, some funders cover the full costs of a journal (Diamond OA) to make it free for readers and authors alike. The OA vision is enthusiastically endorsed and often required by prime funders, including the European Union [1]. In this Commentary, we share some negative experiences highlighting that OA remains subject to aggressive attacks and defamations, often without rationale or evidence [2].

Free online access to peer-reviewed publications is particularly important in public health sciences, given its contribution to evidence-based public health policy and practice. The Swiss School of Public Health (SSPH+) – a not-for-profit foundation assembling a public health faculty virtually across fourteen Swiss universities – shares the vision of OA as a key pillar of open science. In line with the OA strategy of Swiss universities [3] and the Swiss National Science Foundation [4], which aim to achieve 100% OA publishing in Switzerland, SSPH+ decided to run its two journals – namely the International Journal of Public Health (IJPH) and Public Health Reviews (PHR) – as Gold OA journals. Since both journals mainly publish publicly funded research, SSPH+ considered it unethical to continue with the hitherto used hybrid model of IJPH. Under that previous model, the publisher sold access to IJPH through subscriptions while also charging APCs to authors who opted to publish OA. This hybrid model is routinely criticized for using an unfair “double dipping” business model [5], where revenues from subscriptions are amplified by APC fees charged to authors opting for Gold OA.

Following a rigorous tendering process with six publishers, SSPH+ contracted an experienced Gold OA publisher for the provision of publishing services, namely Switzerland-based Frontiers Media. With this transition to OA, SSPH+ achieved full independence from its publisher, including the freedom to drop quotas on the number of annual publications enforced by the previous publisher to protect subscription revenues. SSPH+ was also able to lower APC fees and set up GLOBEQUITY, a fee waiver program for first authors from low and middle-income countries.

However, instead of receiving congratulations for the successful transition to OA, we, the editors and the independent Editorial Management Office, have experienced negative rumors, attacks, and defamations against both IJPH and PHR. The first attack reached SSPH+ in early 2023 when both journals appeared on an anonymous “black list” of supposedly predatory journals [6]. The anonymous leadership of the predatory.org attack placed both journals on a long list of what they considered “predatory journals,” despite the fact that none of the criteria of predatory journals featured on that website applied to the SSPH+ journals. Unable to take legal action against anonymous persons, SSPH+ could intervene only via an anonymous email listed on their website. After several weeks, the SSPH+ journals were removed from this “black list,” which still runs its anonymous defamation campaign against a range of journals, including respected titles in the public health sciences.

Unfortunately, this was just the beginning. Surprisingly, the most recent attacks have come from academic constituencies, namely the Finnish Publication Forum (JUFO). In fall 2024, the Finnish JUFO commission announced that, in 2025, it would “downgrade” a long list of over 270 journals to “grey area Level 0” – a term used for a “blacklist” of journals about which JUFO claims (undefined) “concerns” [7]. This contrasts with the “whitelists” of recommended journals at JUFO’s level 1 (basic), 2, and 3 (top). JUFO acts under the auspice of the Federation of Finnish Learned Societies – a publicly funded authority. Its classification of journals is the point of reference for scientists’ decisions about where to publish and is a key criterion defining the distribution of funding across Finnish universities [8].

JUFO never discussed their “concerns” with our editors or the SSPH+ directorate. The downgrading plan instead reached us in mid-December 2024 via LinkedIn and Retraction Watch. Alarmed, SSPH+ asked the JUFO Chair, at the end of 2024, to abstain from the announced defamation of the more than a hundred dedicated editors of IJPH and PHR [9]. These leading scientists and hundreds of reviewers guarantee an independent, rigorous, and fully transparent double-blinded peer review. Despite the letter, JUFO downgraded both journals in mid-January 2025. Later, SSPH+ was informed about JUFO’s decision and upcoming meetings scheduled for March and May [10]. JUFO did not disclose any rationale nor explain why the downgrading had overridden their own “Level 1” criteria [11]. Both IJPH and PHR fully comply with all seven criteria of the JUFO’s Level 1, and the journals have not undergone changes since the 2012–2024 period, when JUFO listed them at Level 1. A closer look at the classification portal reveals that the classification framework and the JUFO decisions lack any valid, transparent, objective, or replicable methodology. Journal classifications often contradict JUFO’s own objective criteria, such as the seven established requirements for Level 1. Let us share two stunning examples of the obscurities of the JUFO classification:

First, a long-standing “friendly competitor” of IJPH with many “indistinguishable characteristics”, namely the European Journal of Public Health, was listed as Level 2 in 2012–2014, downgraded to Level 1 until 2019, then returned to Level 2 in 2020 [12]. These changes are as irrational as the current situation, where IJPH is placed at Level 0, but EJPH is listed at Level 2. However, both journals fulfill all objective quality criteria of robust scientific publishing. Due to the listing and associated university funding distribution, a 2025 EJPH publication is weighted 30 (!) times higher than an IJPH publication, although scientists often submit to both journals interchangeably. Indeed, the manuscript tracking shows that some papers rejected after peer review by IJPH are later submitted to and published in EJPH. The opposite must also be true, given the similar visions, review modalities, procedures, and rejection rates of these two OA society journals, which even had overlapping Associate Editors over the years. To classify EJPH and IJPH differently raises serious questions about the JUFO classification scheme.

Second, JUFO ignores its own Level 1 criteria, both in downgrading the SSPH+ journals and in endorsing, apparently random, upgrading decisions. Several “Level 3” journals are not compliant with the JUFO Level 1 Criterion Nr. 4, which states: *“The publication channel’s editorial board constitutes of experts, who mainly include researchers working in universities or research institutes.”* The only objective “Level 3” criterion reemphasizes that *“The channels have international authors and readers and the editorial boards are constituted by the leading researcher in the field.”* In fact, this criterion is fully in line with the Guidelines of the International Committee of Medical Journal Editors, which emphasize the relevance of the independence and leading expertise of editors as a condition to guarantee the quality of peer review and to minimize conflicts of interest in editorial decisions [13].

However, JUFO lists instead several journals as “Level 3” that run under a rather different business model. For example, Editors-in-Chief and Handling Editors of some Lancet- or Nature-branded Gold OA journals are neither leading scientists in the field of the journal nor working in universities or research institutions but instead, are staff of the publisher. Hence, editorial decisions are not made by independent scientists, which increases the risk of conflicts between business-oriented criteria, such as collecting citations and scientific quality [14, 15].

In the absence of objective reproducible criteria and assessments, one may wonder about the alternative drivers of JUFO decisions. Do personal opinions, rumors, and the biases of committee members guide classifications? What is the role of friends and colleagues? We do not know, but we realize in our transition to OA that JUFO is not the only academic commission where, apparently, non-evidenced based opinions lead to decisions to the detriment of fair Gold OA journals [16] The JUFO announcement reveals a pattern underlying many defamations against journals published by successful Gold OA publishers including Frontiers Media, the current publisher of IJPH and PHR.

Should SSPH+ return to locking its journals behind paywalls? We do not think so! Instead, science needs engaged and dedicated academics defending rigorous and independent peer-reviewed OA publishing, while standing up against defamations attacking OA publishing. Why do extreme forms of unethical practice exist in today’s publishing world, such as paper mills, fake papers, selling authorship, predatory journals without peer review, and fake reviews? Why are predatory OA APCs paid to publishers who charge three to five times the APC of the fully cost-neutral self-funded SSPH+ journals? These problems only exist because *scientists* decide to support and make use of these unethical options.

Scientists are therefore not only part of the problem, but they are also part of the solution. Authors, editors, reviewers, and members of leading decision-making academic committees should stand up for evidence-based classifications of scientific quality, instead of unfounded ill-defined pseudo-classifications. Wake up, please!
